# Mass spectrometric analysis of chondroitin sulfate-linked peptides

**DOI:** 10.1007/s42485-022-00092-3

**Published:** 2022-10-02

**Authors:** Madan Gopal Ramarajan, Mayank Saraswat, Rohit Budhraja, Kishore Garapati, Kimiyo Raymond, Akhilesh Pandey

**Affiliations:** 1 Department of Laboratory Medicine and Pathology, Mayo Clinic, 200 First ST SW, Rochester, MN 55905 USA; 2 Institute of Bioinformatics, International Technology Park, Bangalore, 560066 India; 3 Manipal Academy of Higher Education (MAHE), Manipal, 576104 Karnataka India; 4 Center for Molecular Medicine, National Institute of Mental Health and Neurosciences (NIMHANS), Hosur Road, Bangalore, 560 029 India; 5 Biochemical Genetics Laboratory, Department of Laboratory Medicine and Pathology, Mayo Clinic, Rochester, MN 55905 USA; 6 Center for Individualized Medicine, Mayo Clinic, Rochester, MN 55905 USA

**Keywords:** Proteoglycans, Glycosaminoglycans, Glycoproteomics, Glycopeptide, HCD, EtHCD

## Abstract

Chondroitin sulfate proteoglycans (CSPGs) are extracellular matrix components composed of linear glycosaminoglycan (GAG) side chains attached to a core protein. CSPGs play a vital role in neurodevelopment, signal transduction, cellular proliferation and differentiation and tumor metastasis through interaction with growth factors and signaling proteins. These pleiotropic functions of proteoglycans are regulated spatiotemporally by the GAG chains attached to the core protein. There are over 70 chondroitin sulfate-linked proteoglycans reported in cells, cerebrospinal fluid and urine. A core glycan linker of 3–6 monosaccharides attached to specific serine residues can be extended by 20–200 disaccharide repeating units making intact CSPGs very large and impractical to analyze. The current paradigm of CSPG analysis involves digesting the GAG chains by chondroitinase enzymes and analyzing either the protein part, the disaccharide repeats, or both by mass spectrometry. This method, however, provides no information about the site of attachment or the composition of linker oligosaccharides and the degree of sulfation and/or phosphorylation. Further, the analysis by mass spectrometry and subsequent identification of novel CSPGs is hampered by technical challenges in their isolation, less optimal ionization and data analysis. Unknown identity of the linker oligosaccharide also makes it more difficult to identify the glycan composition using database searching approaches. Following chondroitinase digestion of long GAG chains linked to tryptic peptides, we identified intact GAG-linked peptides in clinically relevant samples including plasma, urine and dermal fibroblasts. These intact glycopeptides including their core linker glycans were identified by mass spectrometry using optimized stepped higher energy collision dissociation and electron-transfer/higher energy collision dissociation combined with hybrid database search/de novo glycan composition search. We identified 25 CSPGs including three novel CSPGs that have not been described earlier. Our findings demonstrate the utility of combining enrichment strategies and optimized high-resolution mass spectrometry analysis including alternative fragmentation methods for the characterization of CSPGs.

**Supplementary Information:**

The online version contains supplementary material available at 10.1007/s42485-022-00092-3.

## Introduction

Proteoglycans consist of a core protein with one or more glycosaminoglycan chains covalently attached to it. Glycosaminoglycans (GAGs) are heteropolymers of repeating disaccharides—consisting of an amino sugar and a uronic acid—linked to serine residues on a core protein via a linker oligosaccharide (Merry et al. 2022). The most common GAGs are chondroitin sulfate (CS), dermatan sulfate (DS), keratan sulfate, hyaluronan, heparan sulfate (HS) and heparin (Lebrilla et al. 2022). Chondroitin sulfate proteoglycans (CSPGs), dermatan sulfate proteoglycans and heparan sulfate proteoglycans are linked to serine residues through a common core tetrasaccharide linkage (Bella and Danishefsky 1968; Stern et al. 1971; Seno and Sekizuka 1978; Akiyama and Seno 1981; Prydz and Dalen 2000; Mizumoto et al. 2013; Lindahl et al. 2017). Proteoglycans are an integral part of skin and connective tissues and are involved in various physiological processes including cell adhesion, growth and differentiation, signaling, angiogenesis and anti-coagulation and have also been implicated in tumor progression and metastases (Iida et al. 1996; Kastana et al. 2019; Perrimon and Bernfield 2001; Stringer 2006; Wei et al. 2020).

Chondroitin sulfate proteoglycans (CSPGs) are principal components of pericellular and extracellular matrices of connective tissues. CSPGs are composed of anionic GAGs linked to the hydroxyl group of a serine residue on the core protein through a variable oligosaccharide linker, the commonest of which is made up of one glucuronic acid (GlcA), two galactose (Gal) units and a xylose (Xyl) (*β*4GlcA*β*3Gal*β*3Gal*β*4Xyl*β*1-O-Ser), which may be modified by sulfation (Lindahl et al. 2017). The oligosaccharide moiety in chondroitin sulfate is composed of repeating disaccharide units of N-acetylgalactosamine (GalNAc) and D-glucuronic acid. The nascent core protein synthesized in the cytosol by translation is translocated to the lumen of endoplasmic reticulum, where synthesis of the linkage region occurs followed by assembly of chondroitin sulfate chains in the Golgi compartment (Mikami and Kitagawa 2013). Sulfation and/or phosphorylation are the most common modifications found in both linkage regions and chondroitin sulfate chains of CSPGs, though sialylation and fucosylation have been reported (Gomez Toledo et al. 2015). The variable sulfation pattern as well as the length of CS chains determines the biological activities and specific molecular interactions of CSPGs. CSPGs are involved in a wide range of cellular processes, growth factor signaling and inflammation (Klüppel et al. 2005; Mizumoto et al. 2015; Stephenson et al. 2018). In addition, they play a role in the organization of extracellular matrix of the brain and in controlling neuronal growth and plasticity (Maeda et al. 2010; Siebert et al. 2014). Monosulfated moieties on GAGs facilitate binding to cytokines, cell surface receptors and growth factors such as vascular endothelial growth factor (VEGF) (Hirose et al. 2002; Kwok et al. 2012; Mikami and Kitagawa 2013; Zhou et al. 2014; Koike et al. 2015; Shintani et al. 2006).

Several clinical disorders are known to be associated with CSPG synthesis, structure and degradation. In CSPGs, sulfation can occur at C-2 and C-3 positions of GlcA, and the C-4’ and C-6’ positions of GalNAc, thus accounting for 16 possible disaccharide modifications (Wei Poh et al. 2015). Based on the sulfation pattern, the CS chains are classified as monosulfated and disulfated CS. The monosulfated CS chains are CS-A (-GlcAβ1-3GalNAc-4-sulfate-) and CS-C (-GlcAβ1-3GalNAc-6-sulfate-). The disulfated CS chains are CS-B (-GlcA-2-sulfateβ1-3GalNAc-4-sulfate-), CS-D (-GlcA-2-sulfateβ1-3GalNAc-6-sulfate-), CS-E (-GlcAβ1-3GalNAc-4-sulfate-6-sulfate-) and CS-K (-GlcA-3-sulfateβ1-3GalNAc-4-sulfate-) (Nandini and Sugahara 2006; Afratis et al. 2012; Wei Poh et al. 2015; Kastana et al. 2019; Wang et al. 2020). Imbalance in the sulfation pattern of CSPGs has been shown to have a role in autoimmune disorders such as systemic lupus erythematosus and dermatomyositis (du Souich et al. 2009; Kim and Werth 2011). The type of sulfation pattern in CSPGs has been suggested to be a critical factor in the progression of cancer (Theocharis et al. 2006). CSPGs with CS-A sulfation pattern are implicated in metastatic cascade through activation of MMP2 (Iida et al. 2007). CSPGs with 6-O-sulfation are associated with tumor progression, growth and metastasis in hepatocellular carcinoma (Jia et al. 2012) melanoma, osteosarcoma (Cattaruzza et al. 2008; Nikitovic et al. 2008) and other cancers (Asimakopoulou et al. 2008). CSPGs with overexpression of CS-E chains mediate VEGF binding in ovarian adenocarcinomas (Ten Dam et al. 2007). The chondroitin-6-sulfate present in CSPGs binds to lipoproteins, causing accumulation, oxidation and hydrolysis of low-density lipoproteins in the arterial wall leading to development of atherosclerosis (Scuruchi et al. 2020). Mutations in the chondroitin sulfate synthetic machinery/enzymes can lead to connective tissue disorders like Ehlers-Danlos syndrome and skeletal dysplasia and ocular disorders such as congenital corneal stromal dystrophy and Meester–Loeys syndrome (Mizumoto et al. 2013; Meester et al. 2017; Paganini et al. 2019). Mutations in the lysosomal enzymes required to break down the long chains of GAGs lead to a group of metabolic disorders called mucopolysaccharidoses characterized by accumulation of chondroitin sulfate, dermatan sulfate and/or heparan sulfate in connective tissues (Muenzer 2011). These accumulated CSPGs bind to protein tyrosine phosphatase receptors and to Nogo receptors thus inhibiting neuronal growth and axon regeneration in glial scar tissues (Brown et al. 2012; Tran et al. 2018). Several pathogenic microorganisms including viruses (herpes simplex virus, dengue virus, respiratory syncytial virus), bacteria (*Borrelia burgdorferi*) and parasites (*Plasmodium falciparum*) can express proteins that bind to the CS chains of CSPG present on the cell surface and invade the host cells (Jinno and Park 2015).

Analysis of proteoglycans is challenging and involves isolation, enrichment and depolymerization. Proteoglycans from homogenized tissues or cells have been traditionally analyzed by extraction with guanidium hydrochloride followed by anion exchange chromatography or size-exclusion chromatography (Ly et al. 2010). These crude proteoglycans are then processed by treating them with non-specific proteases to generate GAG chains. The glycosaminoglycan attached to the specific serine residues of core protein may also be released by β-elimination followed by Michael addition of dithiothreitol (BEMAD) (Wells et al. 2002). The released GAGs are depolymerized by highly specific lyases to disaccharides or partially depolymerized oligosaccharides whose compositions are analyzed by chromatography, nuclear magnetic resonance spectroscopy or by mass spectrometry (Barroso et al. 2005; Chi et al. 2008; Sisu et al. 2011). Deducing the structural components of disaccharides and location of sulfation groups can provide information about the type of glycosaminoglycan chain isolated. Structural characterization of proteoglycans is a challenging task mainly due to the astounding structural diversity in terms of size, charge and degree of sulfation. Multiple glycan chains can be attached to the same core protein at different sites with differing glycan compositions. Analytical limitations further include low coverage of proteoglycan peptide sequence and significant sulfate losses.

Glycoproteomics strategies involve specific enrichment of glycopeptides followed by liquid chromatography–tandem mass spectrometry (LC–MS/MS) (Saraswat et al. 2021). Based on the chemical nature of proteoglycans, strong anion exchange (SAX) chromatography has been used to optimize their enrichment and analysis to identify CS-linked glycopeptides (Nilsson et al. 2009; Noborn et al. 2015). Our goal was to characterize CSPGs by combining different enrichment strategies in addition to the utility of optimized stepped higher energy collision dissociation (stepped HCD) and electron-transfer/higher energy collision dissociation (EtHCD). Based on this strategy, we analyzed CSPGs in plasma, urine and dermal fibroblasts. We identified 25 CSPGs including 3 novel CSPGs—membrane-associated progesterone receptor component 1, tenascin and collagen alpha–3 (V) chain.

## Results and discussion

**Fig. 1:**
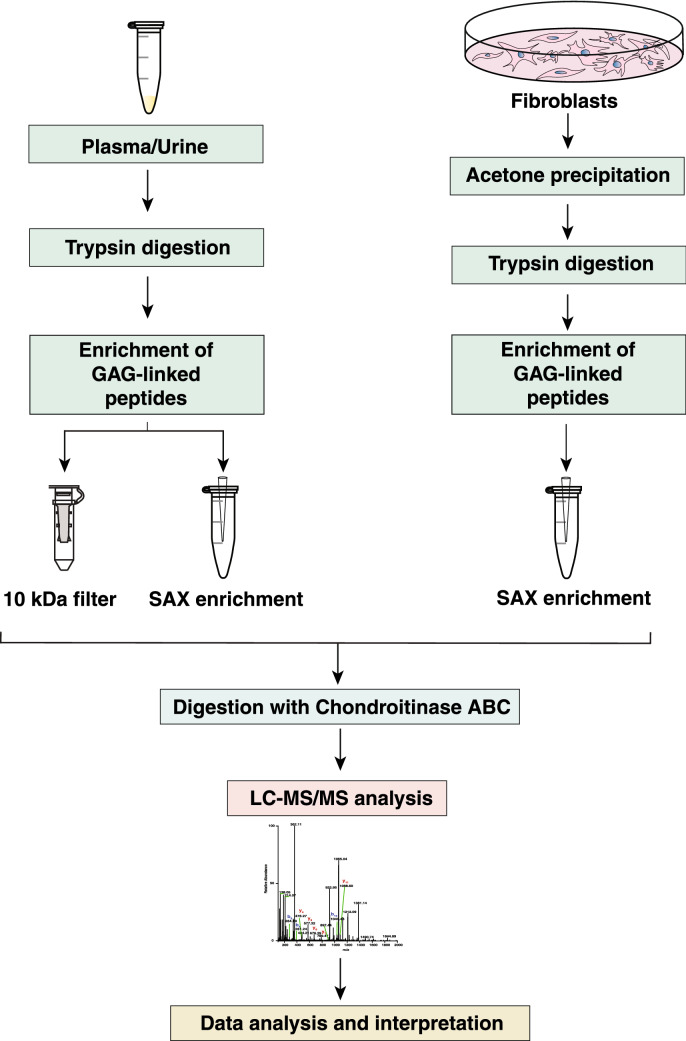
A schematic showing the workflow for enrichment and analysis of intact GAG-linked glycopeptides. The schematic depicts stepwise processing of indicated samples by trypsin digestion, intact glycopeptide enrichment, chondroitinase ABC digestion and LC–MS/MS analysis. Two different enrichment strategies were employed for plasma and urine samples as shown—ultrafiltration (10 kDa MWCO) and strong anion exchange (SAX) for enriching intact chondroitin sulfate (CS) chains attached to tryptic peptides. For fibroblast samples, SAX was employed for enriching the CS-linked peptides. The CS chains obtained from all sample types were digested by chondroitinase ABC enzyme mixture to yield glycopeptides with linker oligosaccharide attached to peptide backbones as indicated

We carried out high-throughput analysis of chondroitin sulfate-linked intact glycopeptides from clinically relevant samples including body fluids such as plasma and urine and dermal fibroblasts. In the following sections, we describe our approach in a detailed stepwise manner (Fig. 1).

### Sample preparation

Blood collected in lithium heparin tubes was centrifuged twice to obtain platelet-poor plasma, which was used for subsequent experiments. Urine was centrifuged to eliminate cells and debris and supernatant was processed for further experiments. Cultured dermal fibroblasts were thoroughly washed with PBS to get rid of residual FBS. All samples were used for measuring protein concertation by BCA assay.

### Enrichment of chondroitin-sulfate-linked glycopeptides

We sought to characterize chondroitin sulfate-linked proteoglycans present in the plasma. After trypsin digestion, we enriched the glycosaminoglycan linked peptides using two different enrichment strategies based on their molecular weight and charge status: filtration using 10 kDa molecular weight cutoff filter (MWCO) and SAX using spin columns. The molecular weight of chondroitin sulfate-linked tryptic glycopeptides is expected to be 20–55 kDa, depending upon the length of the CS chains. Thus, we used a 10 kDa MWCO filter membranes to enrich these glycopeptides. As a complementary enrichment method, we used SAX chromatography to enrich CSPGs as they contain multiple negative charges mainly due to the high degree of sulfation. In this method, glycopeptides were eluted with increasing salt concentrations for sequential enrichment of sulfated proteoglycans. The enriched glycopeptides obtained from the two enrichment strategies were treated with chondroitinase ABC to depolymerize the CS chains. The resulting oligosaccharide-linked chondroitin sulfate glycopeptides were analyzed by mass spectrometry as described below.

### Mass spectrometric analysis

The glycopeptides were reconstituted and samples were analyzed by LC–MS/MS in positive ion mode. Intact glycopeptides were first separated on a reversed-phase C_18_ column by 150-min gradient and fragmented using complementary techniques of stepped high-energy collision dissociation (stepped HCD) and electron-transfer/higher energy collision dissociation (EtHCD).

#### Data analysis

Peptide identification is often hindered by the presence of glycosylation sites reducing the overall sequence coverage achievable by mass spectrometric methods. To improve the peptide sequence coverage of complex proteoglycan, we employed two different enrichment strategies coupled with bioinformatics tools such as GlycReSoft (Klein et al. 2018a, b) and Mascot with specified modifications to allow for glycopeptide identification.

The MS/MS spectra were filtered to look for *m/z* 362.11 oxonium ion which corresponds to the terminal dehydro-disaccharide [∆HexAGalNAc] + . Further, we observed other fragment ions corresponding to tetrasaccharide ([Δ HexAGlcNAcGlcAGal] + ; *m/z* 699.18) and pentasaccharide ([Δ HexAGlcNAcGlcAGalGal] + ; m/z 861.24).

### Profile of proteoglycans from plasma

By enriching the GAG-linked glycopeptides and utilizing both enrichment strategies and dissociation methods, we identified 17 intact glycopeptides from 2 major proteoglycans found in plasma (Table 1).

**Table 1:**
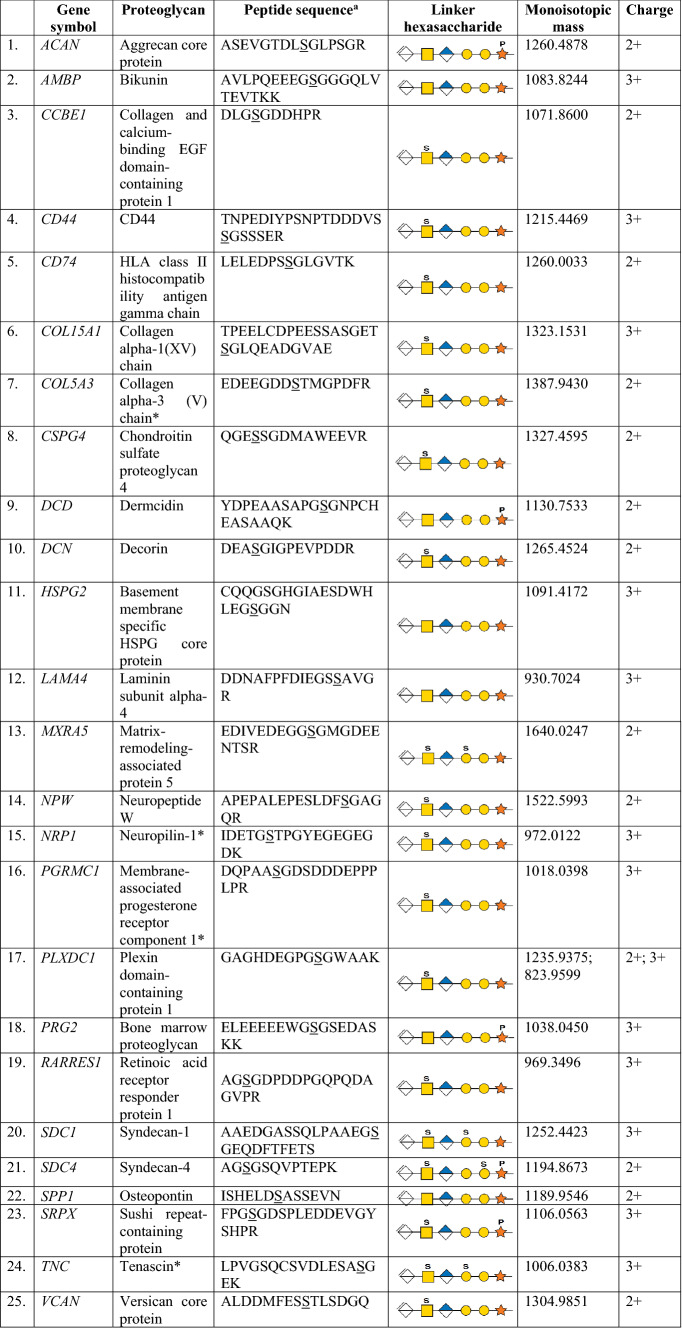
All identified chondroitin sulfate proteoglycans

Chondroitin sulfate proteoglycans are depicted with gene symbols, protein name, peptide sequence, linker hexasaccharide with order of attachment, plausible glycosylation sites, monoisotopic mass and charge of the precursor ion

*Novel chondroitin-sulfate-linked glycopeptides identified, where the previously unreported site is depicted

^a^Underlined serine residues indicate glycan attachment sites

#### Bikunin is the major CSPG in plasma

**Fig. 2:**
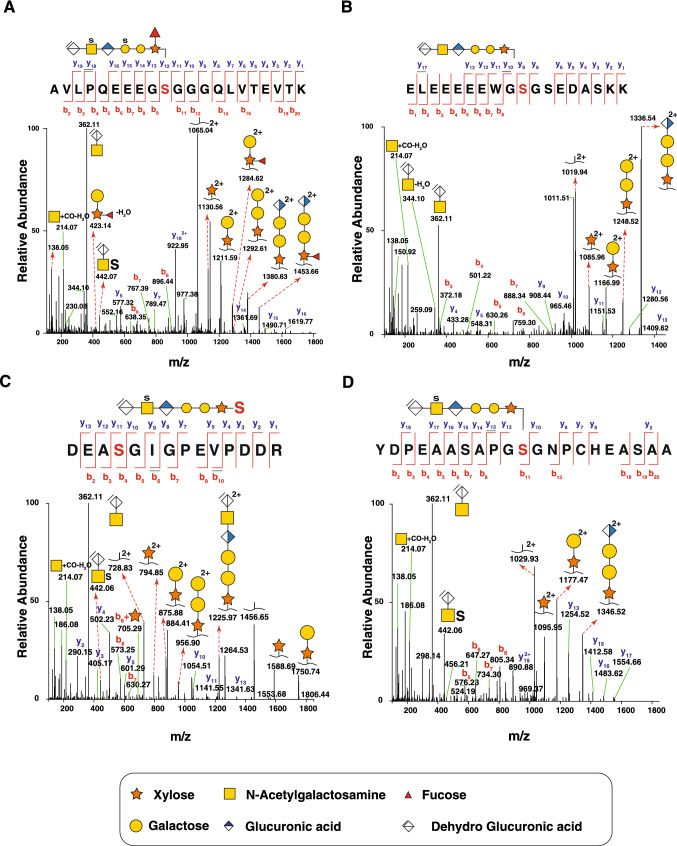
Mass spectrometric identification of chondroitin sulfate-linked proteoglycans. MS/MS spectra of chondroitin sulfate-linked glycopeptides from bikunin (**A**) and bone marrow proteoglycan (**B**) from plasma are shown. The peptides were obtained after enrichment with strong anion exchange chromatography. In bikunin (**A**), the oligosaccharide is composed of [ΔGlcAGalNAcGlcAGalGalXyl] with fucose attached to xylose residue and sulfation of galactose and GalNAc, where Δ denotes dehydroGlcUronic acid. This disulfated fucosylated oligosaccharide linker is attached to Ser-215 on the peptide sequence ^206^AVLPQEEEGSGGGQLVTEVTK^226^. The site of attachment of the oligosaccharide linker to Ser residue on the peptide is colored in red. The b-ions (red) and y-ions (blue) are labeled including the chondroitin sulfate specific oxonium ions—*m/z* 362.11 and *m/z* 214.07. MS/MS spectra of glycopeptide from bone marrow proteoglycan obtained from plasma (**B**) depicted has the hexasaccharide [ΔGlcAGalNAcGlcAGalGalXyl] linked to Ser-62 on the peptide ^53^ELEEEEEWGSGSEDASKK^70^. Representative MS/MS spectra of glycopeptides from decorin (**C**) and dermcidin (**D**) obtained from urine are depicted with the peptide sequence, site of attachment of the oligosaccharide linker (red S), the b-ions (red) and y-ions (blue). The monosaccharide symbols depicted are according to the consortium for functional glycomics (http://www.functionalglycomics.org/static/consortium/Nomenclature.shtml), and the charge states of all fragment ions with charge higher than 1 + are shown

Bikunin is a chondroitin sulfate-linked proteoglycan present in circulation as inter-alpha-trypsin inhibitor light chain of the protein alpha-1-microglobulin/bikunin precursor (AMBP) (Zhuo et al. 2002). Overall, we identified 10 glycopeptides belonging to bikunin from plasma. The glycosite was observed to be on serine preceded by an acidic amino acid and followed by glycine (Seno et al. 1978). The glycopeptide sequence, ^206^AVLPQEEEGSGGGQLVTEVTK^226^, was predominantly observed with attachment of a linker hexasaccharide on serine residue at position 215, with a monoisotopic mass of *m/z* 1094.43; 3 + . This bikunin glycopeptide precursor ion was found in both 800 mM NaCl and 1.6 M NaCl eluates of SAX chromatography. The HCD spectrum revealed intense saccharide oxonium ions in the m/z interval of 100 to 500. The prominent oxonium ions were observed at *m/z* 362.11 and *m/z* 380.12 which are specific to the presence of chondroitin sulfate glycopeptides. Chondroitinase ABC extracts a water molecule from the C4-C5 of the terminal glucuronic acid leading to loss of stereoisomerism at C4 and C5, forming [∆HexAGalNAc] + , which is observed as a prominent ion at *m/z* 362.11 (Noborn et al. 2015). Due to fragmentation of HexNAc B-ion *m/z* 214.09, saccharide oxonium ions at *m/z* 204.09, *m/z* 186.08, *m/z* 138.05 and *m/z* 126.05 were also observed. Further, the presence of peptide + xylose (m/z 1130.56; 2 +) followed by peptide + xylose + galactose (m/z 1211.59; 2 +) and others were observed (Fig. 2A). This permitted us to deduce the sequential loss of sugar moieties from the hexasaccharide linker attached to Ser residue on the peptide. We were able to identify a glycoform carrying a phosphate moiety on xylose residue, detected in plasma samples in both dissociation modes (Gomez Toledo et al. 2015). The various saccharide linkers attached to serine are provided in the supplementary Table S1. One of the linker molecules is fucosylated and bisulfated hexasaccharide chain. In human chondroitin sulfate proteoglycans, the fucose modification is notably seen on the xylose residue close to the site of attachment to serine residue of the protein. This is similar to core fucosylation found on the innermost GlcNAc residues of N-glycans (Vainauskas et al. 2016).

#### Bone marrow proteoglycan

Bone marrow proteoglycan (PRG2), a major constituent of eosinophil granules, is a proinflammatory protein with potent cytotoxic and anti-helminthic activity (Shikata et al. 1993). Bone marrow proteoglycan is also known as major basic protein (MBP) and is synthesized in the cells as a pre-pro form. The pre-pro form is converted to pro-MBP by proteases and further digested to form MBP. Four mucin type O-glycan sites, one N-glycan site and one O-linked glycosaminoglycan site have been reported on this protein (Shikata et al. 1993), with the presence of chondroitin sulfate-linked glycan at Ser-62 (Oxvig et al. 1994). We detected two glycopeptides with two different hexasaccharide linkers on the peptide sequence—^53^ELEEEEEWGSGSEDASKK^70^. This particular glycopeptide sequence has been reported in urine (Noborn et al. 2015), but here we report these glycopeptides in the plasma for the first time using high-resolution mass spectrometry. The spectrum showed saccharide oxonium ions, peptide backbone fragmentation (3 consecutive b- and y-ions) and peptide + saccharide ions (peptide + xylose, peptide + xylose + galactose, etc.) (Fig. 2B).

### Chondroitin sulfate-linked proteoglycans detected in urine

Our methods detected other chondroitin sulfate-linked proteoglycans: decorin, dermicidin, plexin domain-containing protein-1, osteopontin and collagen alpha-1(XV). Overall, we detected 75 glycopeptides on 36 glycosites from 15 proteoglycans (Table 1).

Decorin is a small leucine-rich proteoglycan whose core protein is linked to either chondroitin sulfate or dermatan sulfate chains depending on the tissue. The CS chain is attached to Ser-34 on the N-terminus of the protein. Characterization of this proteoglycan is further difficult because of isobaric nature of GlcA in CS chains and iduronic acid in DS chains. Decorin CS is composed of repeating disaccharide units of D-N-Acetylgalactosamine and d-glucuronic acid residues which are further modified. The d-glucuronic acid residues are epimerized to l-iduronic acid by the enzyme C5-epimerase in collagen fibers. On the other hand, the dermatan sulfate-linked decorin can be sulfated at C-4 and C-6 of D-GalNAc adjacent to the l-iduronic acid or at C-2 of l-iduronic acid. In our study, we found 3 CS-linked glycopeptides at a single glycosite (Ser-34) on decorin. The glycan composition of the three glycopeptides is provided in supplementary Table S1. The peptide ^31^DEASGIGPEVPDDR^44^ (Fig. 2C) follows the general rule for chondroitin sulfate linker attachment, i.e., the presence of acidic residues (D, E) on the N-terminal and followed by Ser-Gly sequence, which is a consensus site for GAG attachment. One of them is composed of phosphorylated xylose, which is a stoichiometric feature seen in mature proteoglycans, indicating that proteoglycan biosynthesis may be at a balance between kinases and phosphorylases (Wen et al. 2014).

Dermcidin is an antimicrobial protein secreted by eccrine sweat glands in humans (Na et al. 2019). The antimicrobial peptide (AMP) domain containing 48 amino acids are proteolytically processed, secreted into sweat and have antimicrobial properties (Schittek 2012). However, there is another putative product of this protein, which forms the peptide core of proteolysis inducing factor (PIF). PIF is a glycosylated cachectic factor with a molecular weight of 24 kDa. It has been shown that the chondroitin sulfate hexasaccharide is linked to Ser-30 on the peptide ^20^YDPEAASAPGSGNPCHEASAAQK^42^ (Fig. 2D). We detected seven glycopeptides with two possible glycosites. In addition to the reported Ser-30 glycosite, we found another site at Ser-38.

#### 2-*O*-Phosphorylation of xylose residue

Phosphorylation of the xylose residue at C2 is one of the key modifications observed in the linkage hexasaccharides in both CS and HS GAG chains (Oegema et al. 1984; Fransson et al. 1985; Moses et al. 1999). This modification is carried out by a Golgi-resident kinase, FAM20B (family with sequence similarity 20 B) (Koike et al. 2009). The 2-O-phosphorylation of xylose is essential for efficient transfer of glucuronic acid residue to the phosphorylated trisaccharide linker. A previous study (Moses et al. 1999), focused on deciphering the biosynthetic mechanisms of the linker trisaccharide on decorin, revealed rapid dephosphorylation of xylose following addition of the first glucuronic acid residue. Although, this transient phosphorylation of xylose residue is a known prerequisite for elongation of the repeating disaccharides in CS and HS chains, some mature forms retain the phosphate on xylose (Tone et al. 2008). We found hexasaccharide linkers with intact 2-O-phosphorylated xylose in bikunin, bone marrow proteoglycan, collagen and calcium-binding EGF domain-containing protein 1, plexin domain-containing protein 1, decorin, HLA class II histocompatibility antigen gamma chain, osteopontin, laminin subunit alpha-4, membrane-associated progesterone receptor component 1, collagen alpha-1(XV) chain and CD44 antigen.

### Cellular CSPG profile

**Fig. 3:**
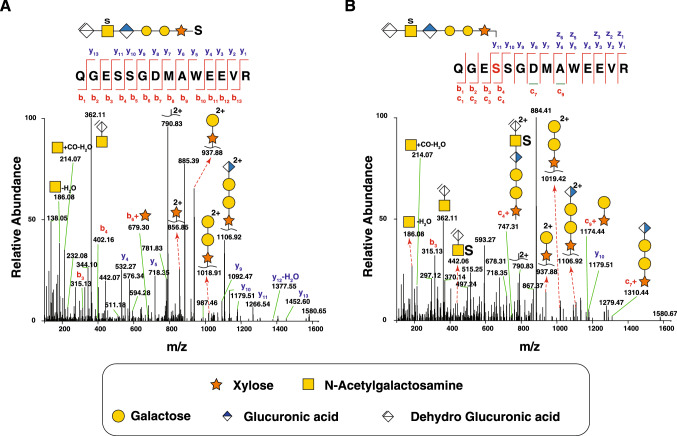
MS/MS fragmentation of a CS-linked glycopeptide using HCD and EtHCD. The complete HCD spectrum (**A**) and EtHCD spectrum (**B**) of the [M + 3H]^3+^ precursor ion at m/z 885.3063 of chondroitin sulfate proteoglycan 4 (CSPG4) is depicted with glycopeptide fragments, saccharide oxonium ions as well as b- and y-ions. The b- and y-ions are depicted in the HCD spectrum (**A**) along with chondroitin sulfate specific ions at *m/z* 362.11 and *m/z* 214.07. The EtHCD spectrum (**B**) shows c- and z-ions in addition to the c-ion + xylose which indicates the attachment of Xylose to Ser-995 on the peptide ^991^QGESSGDMAWEEVR^1004^. The presence of c_4_ + [ΔGlcAGalNAcGlcAGalGalXyl] at *m/z* 747.31 (z = 2 +), confirms the site of attachment of the glycan to the Ser residue on the peptide. Δ denotes dehydroGlcUronic acid. The charge states of all fragment ions with a charge higher than 1 + are shown

To obtain an overview of CSPGs at the cellular level, we also examined GAG-linked peptides using SAX enrichment of fibroblast lysates. We identified 184 glycopeptides from 16 CSPGs including versican, CD44 antigen, decorin, chondroitin sulfate proteoglycan 4 and novel proteoglycans like tenascin-C and collagen alpha-3 (V) chain (Table 1). In addition, we were able to identify novel glycosites and glycan compositions on previously reported CSPGs. We have identified two glycopeptides from CSPG4 proteoglycan with peptide sequence being QGESSGDMAWEEVR. This peptide contains two serine residues which can be potential attachment site for chondroitin sulfate chains. We compared the fragmentation pattern of one of these glycopeptides (m/z 885.3, z = 3) by HCD and EtHCD fragmentation (Fig. 3A,B respectively). In HCD spectrum, 7 y- and 3 b-ions were matched (Fig. 3A) and in EtHCD 2 b-, 9 y-, 2 c-, and 9 z-ions were matched (Fig. 3B). In HCD fragmentation, the site was ambiguous as confirmatory fragments (b4/5 + Xyl or b4/5 + linker oligosaccharide) were not found. While in EtHCD fragmentation, the site could be inferred because c4 ion attached with intact linker oligosaccharide was found, while z10 was found without intact linker oligosaccharide. The bond between serine and chain initiating xylose is an O-glycosidic bond, which can undergo elimination in HCD (Riley et al. 2020; Mao et al. 2021) and peptides which contain two proximal serine residues, EtHCD can be beneficial for localizing the site unambiguously. However, in many cases, we did find evidence of site localization from HCD data, which is likely due to the fact that we employed a stepped collision energy strategy that has also proven useful previously (Noborn et al. 2015; Nikpour et al. 2021). When stepped collision energy is employed, every precursor is fragmented with three different specified collision energies and a composite MS/MS spectrum is reported. At very low energy (NCE = 15) only part of glycan is fragmented and some relatively lower intensity site confirming fragments (Y-type ions) are observed (Riley et al. 2020), while higher energy provides peptide backbone b and y ions.

### Novel proteoglycans/glycopeptides

#### Membrane-associated progesterone receptor component 1 (PGRMC1)

**Fig. 4:**
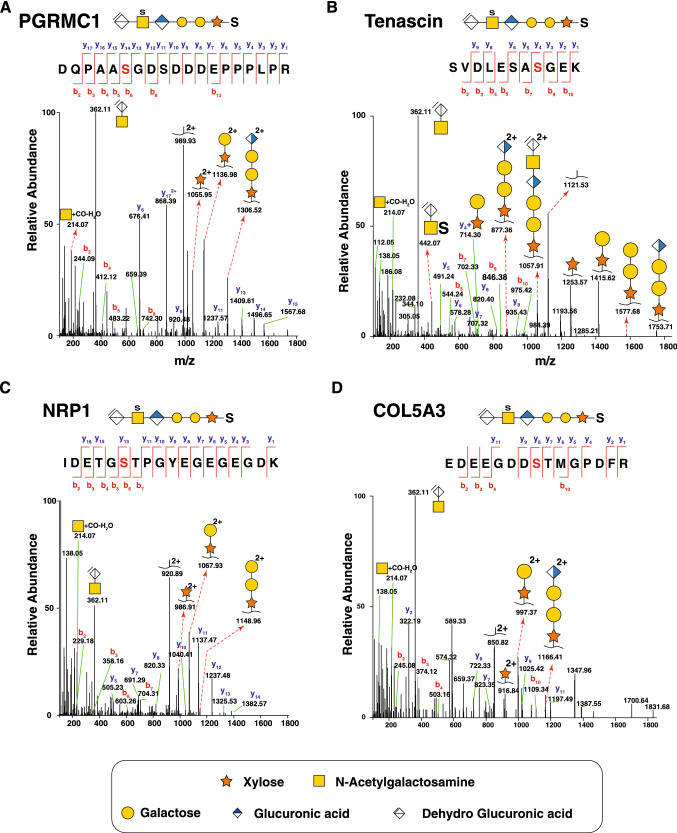
Identification of novel proteoglycans. MS/MS spectra of novel CS-linked proteoglycans along with corresponding hexasaccharide linkers including glycosite on the indicated peptide are shown for membrane-associated progesterone receptor component 1 (PGRMC1) (**A**) detected in urine and for tenascin (TNC) C (**B**) and neuropilin-1 (NRP1) (**C**) detected in fibroblasts. The charge states of all fragment ions with a charge higher than 1 + are shown

Membrane-associated progesterone receptor component 1 is a non-classical progesterone receptor belonging to the b5-like heme/steroid-binding protein family which includes Membrane-associated progesterone receptor component 2, Neudesin and Neuferricin (Ryu et al. 2017; Peterson et al. 2018). This protein acts as a chaperone to transfer heme, cholesterol and steroids, in addition to mediating progesterone signaling and steroid synthesis (Cahill et al. 2017). This protein is not annotated to be glycosylated in UniProt and we report it for the first time to have a CS chain attachment site. We describe a novel CS-linked glycopeptide with CS being linked to Ser-54 on the peptide ^49^DQPAASGDSDDDEPPPLPR^67^ (Fig. 4A). CSPG specific oxonium ions at *m/z* 362.11 and *m/z* 214.09 were detected. In addition, fragmentation of HexNAc B-ion *m/z* 214.09, the saccharide oxonium ions generated at *m/z* 204.09, *m/z* 186.08, *m/z* 138.05 and *m/z* 126.05, were manually confirmed for the glycopeptides. In addition, the peptides + xylose (*m/z* 1055.95) and peptide + XylGal (m/z 1136.98) was also found.

#### Tenascin-C

Tenascin-C is a glycoprotein expressed in dense connective tissues (tendons, ligaments), smooth muscle, stem cells of brain and bone marrow. It is transiently expressed during organ morphogenesis (Chiquet-Ehrismann and Tucker 2011). Tenascin-C regulates adhesion of cells, cellular migration and growth. Tenascin-C binds to fibronectin, thereby regulating cell adhesion. Tenascin-C and fibronectin are similar in size and are often coexpressed (Chiquet-Ehrismann et al. 1988; Orend and Chiquet-Ehrismann 2000; Chiquet-Ehrismann and Chiquet 2003). Tenascin-C expression is increased in inflammatory conditions like psoriasis, lung fibrosis, asthma, inflammatory bowel disease, autoimmune myocarditis, atherosclerosis, IgA nephropathy and primary sclerosing cholangitis. Here, we identified a novel chondroitin sulfate-linked glycopeptide from tenascin-C, (^64^SVDLESASGEK^75^), which contains two plausible glycosites and three hexasaccharide linkers (Fig. 4B). Tenascin-C was detected in fibroblasts in both 800 mM and 1.6 M NaCl eluates.

#### Neuropilin-1 (NRP1)

Neuropilins bind to semaphorin-3A, PlGF-2 (an isoform of placental growth factor), VEGF 165 (isoform of VEGFA) and VEGF-B186 (He and Tessier-Lavigne 1997; Teesalu et al. 2009; Gagnon et al. 2000). Neuropilin-1 (NRP1) interacts with ATP-binding cassette B8 and regulates mitochondrial iron transport (Issitt et al. 2019) NRP1 affects cell survival, migration and angiogenesis. NRP1 binds to a ligand vascular endothelial growth factor (VEGF) thereby regulating VEGF-induced angiogenesis. Neuropilin-1 is also known to act as a host factor for human coronavirus (SARS-CoV-2) infection (Cantuti-Castelvetri et al. 2020). NRP1 has previously been reported to have chondroitin sulfate/ heparan sulfate chains linked to Ser-612 (Shintani et al. 2006). Here, we report an additional novel CS-linked glycopeptide, ^824^IDETGSTPGYEGEGEGDK^841^, with glycosylation at Ser-829 (Fig. 4C). The presence of saccharide oxonium ions at *m/z* 362.11 ([∆HexAGalNAc]^+^) and characteristic HexNAc oxonium ions confirm this sequence to be a glycopeptide. The presence of peptide + XylGalGal∆HexA (m/z 1236.97; 2 +) followed by sequential loss of dehydrated hexuronic acid, two galactose and xylose moieties confirm the presence of linker-saccharide chain on this peptide sequence.

#### Collagen type V alpha-3 chain

Collagen type V alpha-3 chain (COL5A3), a low abundance fibrillar collagen regulates the assembly of type I and type V collagen heterotypic fibers. COL5A3 is a trimer composed of two α1 (V) and one α2 (V) chains. N-glycosylation at site Asn-102 and Asn-141 has been previously reported on COL5A3 (Chen et al. 2009). COL5A3 binds to heparin and is a structural component of the extracellular matrix. COL5A3 interacts through glypican-1, a cell surface heparan sulfate proteoglycan attached to the extracellular surface by a glycophosphatidylinositol anchor (Fico et al. 2011). Glypican-1 is a mitogen required for cell cycle progression (Qiao et al. 2016). We detected a chondroitin sulfate hexasaccharide linker attached to Ser-349 on the peptide sequence ^342^EDEEGDDSTMGPDFR^356^ (Fig. 4D).

## Conclusions

Analysis of CSPGs is technically challenging owing to several factors including inefficient isolation, non-standardized mass spectrometric parameters and less optimal database search tools enabling true high throughput. CSPGs are biologically important molecules with lesser-known functions mainly due to challenging analysis. Here, we employed a multipronged approach—enrichment by complementary methods, analysis of intact glycopeptides by efficient fragmentation methods and an integrative approach for data analysis. We report 25 intact CSPGs including 3 reported for the first time achieving deeper coverage and throughput. After testing the two enrichment strategies, we found that SAX performs better than 10 kDa cutoff filters as significantly more CSPGs were identified using SAX enrichment (Supplementary table S2). We show that stepped collision energy is helpful in sequencing intact GAG-linked glycopeptides as has previously been shown for N-glycopeptides. Several novel proteoglycans such as tenascin-C, membrane-associated progesterone receptor component 1 and collagen type V alpha-3 chain were identified and EtHCD improved localization of some of the sites in glycopeptides, reiterating the importance of alternative fragmentation methods. These methods can be employed to gain insights into human proteoglycome to uncover new biology in diverse sample types.

## Methods

Lithium-heparin anticoagulated control blood samples were used to obtain plasma by double centrifugation and a pool was made by combining equal volumes. About 10 ml of urine was collected from apparently healthy individuals. We procured fibroblasts of apparently healthy individuals (GM05381, GM05399, GM00038, GM08680) from Coriell Institute for Medical Research, New Jersey, USA. This study was approved by the Institutional Review Board at Mayo Clinic (approval number IRB19-004,317).

### Cell culture

Fibroblasts were cultured in MEM alpha medium with 15% fetal bovine serum and 1% non-essential amino acids. Cells were maintained in CO_2_ incubator with 5% CO_2_ level. Once the cells reached 85–90% confluency, the cells were grown in serum free MEM alpha, no phenol red medium for 12 h. After 12 h, the cells were harvested with 1 × modified RIPA buffer (50 mM Tris–HCl, pH 7.4, 150 mM NaCl, 1 mM EDTA, 1% Nonidet P-40, 0.25% sodium deoxycholate and 1 mM sodium orthovanadate) without SDS, after washing with phosphate buffered saline. The cells were stored at -80 °C till further analysis.

### Sample processing and trypsin digestion

The cells were lysed using probe sonication (Branson Sonifier SFX150) at 40% amplitude for 10 s, three cycles, at intervals of 5 min on ice. The lysates were centrifuged at 10,000×*g* for 10 min and the supernatant was used for protein estimation. Protein concentration in plasma, urine and fibroblast lysates was estimated by BCA assay. Aliquots of 4 mg protein from plasma, urine and fibroblast lysates were taken for the experiments. The plasma and urine aliquots were taken in a microcentrifuge tube and were dried in a speed-vacuum concentrator (Savant, Thermo Scientific). The dried pellets were dissolved in 50 μl of 8 M urea in 50 mM triethylammonium bicarbonate buffer (TEAB), pH 8.5. Fibroblast lysates containing 4 mg protein was aliquoted into a 50 ml falcon tube and diluted 10 times with ice-cold acetone. After vortexing vigorously for 10 s, the sample was incubated at -20 °C for 2 h and centrifuged at 14,000×*g* for 20 min. The supernatant was discarded and the pellet was dissolved in 100 µl of 8 M urea in 50 mM TEAB buffer, pH 8.5. Dithiothreitol (Sigma) was added to the sample at a final concentration of 10 mM and incubated at 37 °C for 45 min with mild shaking. The sample was cooled to room temperature (RT) and iodoacetamide (Sigma) was added at a final concentration of 40 mM and incubated for 15 min in the dark at RT. The sample was subsequently diluted 10 times with 50 mM TEAB buffer, pH 8.5 and sequencing-grade trypsin was added to a final amount of 1:50 (trypsin:total protein, w:w) for plasma and urine; and 1:20 (trypsin:total protein, w:w) for fibroblast lysates. The mixture was incubated overnight at 37 °C with mild shaking. Next day, the peptide mixture was enriched for glycosaminoglycan (GAG)-linked peptides.

### Enrichment of GAG-substituted peptides

The trypsin-digested samples were divided equally to be enriched with two different strategies, namely filtration with 10 kDa molecular weight cutoff filters (MWCO) and strong anion exchange chromatography (SAX).

#### Enrichment using filtration with 10 kDa MWCO

The trypsin-digested samples were acidified with 1% trifluoroacetic acid to inactivate the trypsin. The samples were applied to 10 kDa MWCO filters (Amicon Ultra—0.5, Millipore Sigma) and centrifuged at 14,000 × g for 15 min at RT. The filters were washed with 500 µl of wash buffer (100 mM Tris, 50 mM NaCl, 10 mM MgCl_2_, 60 mM sodium acetate, pH 8.0) for three times. The retentate containing GAG-linked peptides present in the filter was inverted into a fresh collection tube and centrifuged at 1,000 x g for 2 min. The retentate was processed for depolymerization with chondroitinase ABC.

#### Enrichment using strong anion exchange chromatography

We followed previously described protocol, for enrichment of GAG-substituted peptides using SAX (Noborn et al. 2015). The trypsin-digested samples were acidified with binding buffer (50 mM sodium acetate, 200 mM NaCl, pH 4.0). The GAG-linked peptides were enriched using SAX TopTips (POROS strong anion exchanger TopTip TT2PSA, Glygen). The SAX tips were conditioned with 50 µl binding buffer (50 mM sodium acetate, 200 mM NaCl, pH 4.0), centrifuged at 700×*g* for 1 min. Sample was added on to the tips and washed with binding buffer. The enriched glycopeptides were eluted sequentially with three buffers of increasing NaCl concentrations and pH—1) 50 mM sodium acetate, 400 mM NaCl, pH 4.0; 2) 50 mM Tris–HCl, 800 mM NaCl, pH 8.0; and 3) 50 mM Tris–HCl, 1.6 M NaCl, pH 8.0. For the wash and elution steps, the tips were spun at 1000×*g* for 2 min. The eluates from 800 mM NaCl and 1.6 M NaCl elution were desalted using a PD Miditrap G25 columns (Cytiva). The desalted fractions were digested with chondroitinase ABC.

#### Digestion with chondroitinase ABC

Chondroitinase ABC (EC 4.2.2.20) (C3667, Sigma Aldrich) was reconstituted with digestion buffer (100 mM Tris–HCl, 50 mM NaCl, 10 mM MgCl_2_, 60 mM sodium acetate, pH 8.0). Two units of Chondroitinase ABC were added to the enriched GAG-substituted peptides. The mixture was incubated overnight at 37 °C with shaking at 750 rpm on a thermomixer. The following day, the peptides were acidified with 1% trifluoroacetic acid and cleaned up with C_18_ tips (TopTip, Glygen) according to manufacturer’s instructions. The eluate from C_18_ tips (50% acetonitrile) was dried at 35 °C in a speed-vacuum concentrator (Savant, Thermo Scientific).

### Liquid chromatography–tandem mass spectrometry (LC–MS/MS)

LC–MS/MS parameters used have been published previously and were used with the following modifications for the current study (Mun et al. 2020; Saraswat et al. 2021). The dried peptides were reconstituted in 0.1% formic acid and were analyzed on an Orbitrap Eclipse Tribrid mass spectrometer connected online to Dionex RSLC3000 liquid chromatography system (Thermo Fisher Scientific). An EASY-Spray column (75 µm × 50 cm, PepMap RSCL C_18_, Thermo Fisher Scientific) packed with 2 μm C_18_ particles was used as a separating device and the column temperature was maintained at 50 °C. Solvent A was 0.1% formic acid in water and solvent B 0.1% formic acid in acetonitrile. Injected peptides were trapped on a trap column (100 mm × 2 cm, Acclaim PepMap100 Nano-Trap, Thermo Fisher Scientific) at a flow rate of 20 µl/min. All samples were analyzed by LC–MS/MS in HCD fragmentation mode as well as EtHCD mode with runs being 150 or 155 min at a flow rate of 300 nl/min. The gradient used for separation was as follows: equilibration at 3% solvent B from 0 to 4 min, 3% to 25% solvent B from 4 to 100 min, 25% to 40% solvent B from 100 to 115 min, 40% to 95% solvent B from 115 to 124 min followed by equilibration for next run at 3% solvent B for 5 min. Ionization of eluting peptides was performed using an EASY-Spray source kept at an electric potential of 2.2 kV. All experiments were done in DDA mode with top 15 ions isolated at a window of 1.2 m/z and default charge state of + 2. Only precursors with charge states ranging from + 2 to + 7 were considered for MS/MS events. Stepped collision energy was applied to fragment precursors at normalized collision energies of 15, 25, and 40. MS precursor mass range was set to 400–2000 m/z and 100–2000 for MS/MS. Automatic gain control for MS and MS/MS were 8 × 10^5^ and 2 × 10^5^ and injection time to reach AGC were 50 ms and 200 ms, respectively. Exclude isotopes feature was set to “ON” and 30 s dynamic exclusion was applied. Data acquisition was performed with option of lock mass (*m/z* 441.12002) for all data. For EtHCD runs, all parameters were same except ETD was used as the fragmentation method along with supplemental activation and calibrated charge-dependent ETD parameters.

### Data analysis

Raw files were processed using Proteome Discoverer 2.5 software suite and Mascot. The searches were conducted against UniProt human reviewed protein sequences (20,432 entries). Trypsin specificity was set to semi-tryptic with 3 missed cleavages allowed. Precursor tolerance was set to 5 ppm and fragment tolerance to 10 ppm. Cysteine carbamidomethylation was set as a fixed modification and oxidation of methionine, protein N-terminal acetylation, were set as variable modifications. In addition, variable modifications corresponding to chondroitin sulfate hexasaccharide [ΔGlcAGalNAcGlcAGalGalXyl] on Ser residues were defined as follows: without sulfate (C_37_H_55_NO_30_, 993.2809 Da), with one sulfate (C_37_H_55_NO_33_S, 1073.2377 Da) and two sulfate residues (C_37_H_55_NO_36_S_2_, 1153.1945 Da). The results were filtered at 1% FDR at peptide, glycan and glycopeptide levels. Glycopeptide PSM lists were reduced to unique glycopeptides per search for further manual analysis. Individual spectra were manually verified for quality and oxonium ions.

### Glycopeptide analysis using GlycReSoft

GlycReSoft is an open-source software for analysis of glycomics and glycoproteomics LC–MS/MS data (Klein et al. 2018a, b). The raw files were de-isotoped and charge states deconvoluted from MS1 and MS2 scans. The glycomics search space was constructed using the 20 linker-saccharide compositions described previously (Klein et al. 2018a, b). We manually compiled a list of 288 proteoglycans from UniProt and list of previously published chondroitin sulfate proteoglycans (Toledo et al. 2020). The glycopeptide search space for each sample was constructed using the mzML file. The preprocessed mass spectra were searched against the associated database for glycopeptide identifications using an error tolerance of 10 ppm for the precursor ions and 20 ppm for product ions. The identified glycopeptides with q-value 0.05 at spectrum-level were compiled.

## Supplementary Information

Below is the link to the electronic supplementary material.

Supplementary file1 (PDF 144 KB)

Supplementary file2 (XLSX 38 KB)

## Abbreviations

CSPG: Chondroitin sulfate proteoglycans
GAG: Glycosaminoglycan
HCD: High-energy collision-induced dissociation
EtHCD: Electron-transfer/higher energy collision dissociation
SAX: Strong anion exchange chromatography

## Declarations

### Data deposition

The mass spectrometry glycoproteomics data have been deposited to the ProteomeXchange Consortium via the PRIDE partner repository with the dataset identifier PXD029400.

## References

[CR1] Afratis N, Gialeli C, Nikitovic D, Tsegenidis T, Karousou E, Theocharis AD, Pavão MS, Tzanakakis GN, Karamanos NK (2012). Glycosaminoglycans: key players in cancer cell biology and treatment. FEBS J.

[CR2] Akiyama F, Seno N (1981). Linkage regions between dermatan polysulfates and peptides. Biochim Biophys Acta.

[CR3] Asimakopoulou AP, Theocharis AD, Tzanakakis GN, Karamanos NK (2008). The biological role of chondroitin sulfate in cancer and chondroitin-based anticancer agents. In Vivo.

[CR4] Barroso B, Didraga M, Bischoff R (2005). Analysis of proteoglycans derived sulphated disaccharides by liquid chromatography/mass spectrometry. J Chromatogr A.

[CR5] Bella A, Danishefsky I (1968). The dermatan sulfate-protein linkage region. J Biol Chem.

[CR6] Brown JM, Xia J, Zhuang B, Cho KS, Rogers CJ, Gama CI, Rawat M, Tully SE, Uetani N, Mason DE, Tremblay ML, Peters EC, Habuchi O, Chen DF, Hsieh-Wilson LC (2012). A sulfated carbohydrate epitope inhibits axon regeneration after injury. Proc Natl Acad Sci U S A.

[CR7] Cahill MA, Medlock AE (2017). Thoughts on interactions between PGRMC1 and diverse attested and potential hydrophobic ligands. J Steroid Biochem Mol Biol.

[CR8] Cantuti-Castelvetri L, Ojha R, Pedro LD, Djannatian M, Franz J, Kuivanen S, van der Meer F, Kallio K, Kaya T, Anastasina M, Smura T, Levanov L, Szirovicza L, Tobi A, Kallio-Kokko H, Österlund P, Joensuu M, Meunier FA, Butcher SJ, Winkler MS, Mollenhauer B, Helenius A, Gokce O, Teesalu T, Hepojoki J, Vapalahti O, Stadelmann C, Balistreri G, Simons M (2020). Neuropilin-1 facilitates SARS-CoV-2 cell entry and infectivity. Science.

[CR9] Cattaruzza S, Nicolosi PA, Perris R (2008). Proteoglycans in the control of tumor growth and metastasis formation. Connect Tissue Res.

[CR10] Chen R, Jiang X, Sun D, Han G, Wang F, Ye M, Wang L, Zou H (2009). Glycoproteomics analysis of human liver tissue by combination of multiple enzyme digestion and hydrazide chemistry. J Proteome Res.

[CR11] Chi L, Wolff JJ, Laremore TN, Restaino OF, Xie J, Schiraldi C, Toida T, Amster IJ, Linhardt RJ (2008). Structural analysis of bikunin glycosaminoglycan. J Am Chem Soc.

[CR12] Chiquet-Ehrismann R, Chiquet M (2003). Tenascins: regulation and putative functions during pathological stress. J Pathol.

[CR13] Chiquet-Ehrismann R, Tucker RP (2011). Tenascins and the importance of adhesion modulation. Cold Spring Harb Perspect Biol.

[CR14] Chiquet-Ehrismann R, Kalla P, Pearson CA, Beck K, Chiquet M (1988). Tenascin interferes with fibronectin action. Cell.

[CR15] du Souich P, García AG, Vergés J, Montell E (2009). Immunomodulatory and anti-inflammatory effects of chondroitin sulphate. J Cell Mol Med.

[CR16] Fico A, Maina F, Dono R (2011). Fine-tuning of cell signaling by glypicans. Cell Mol Life Sci.

[CR17] Fransson LA, Silverberg I, Carlstedt I (1985). Structure of the heparan sulfate-protein linkage region. Demonstration of the sequence galactosyl-galactosyl-xylose-2-phosphate. J Biol Chem.

[CR18] Gagnon ML, Bielenberg DR, Gechtman Z, Miao HQ, Takashima S, Soker S, Klagsbrun M (2000). Identification of a natural soluble neuropilin-1 that binds vascular endothelial growth factor: In vivo expression and antitumor activity. Proc Natl Acad Sci U S A.

[CR19] Gomez Toledo A, Nilsson J, Noborn F, Sihlbom C, Larson G (2015). Positive Mode LC-MS/MS Analysis of Chondroitin Sulfate Modified Glycopeptides Derived from Light and Heavy Chains of The Human Inter-α-Trypsin Inhibitor Complex. Mol Cell Proteomics.

[CR21] He Z, Tessier-Lavigne M (1997). Neuropilin is a receptor for the axonal chemorepellent Semaphorin III. Cell.

[CR22] Hirose J, Kawashima H, Swope Willis M, Springer TA, Hasegawa H, Yoshie O, Miyasaka M (2002). Chondroitin sulfate B exerts its inhibitory effect on secondary lymphoid tissue chemokine (SLC) by binding to the C-terminus of SLC. Biochim Biophys Acta.

[CR23] Iida J, Meijne AM, Knutson JR, Furcht LT, McCarthy JB (1996). Cell surface chondroitin sulfate proteoglycans in tumor cell adhesion, motility and invasion. Semin Cancer Biol.

[CR24] Iida J, Wilhelmson KL, Ng J, Lee P, Morrison C, Tam E, Overall CM, McCarthy JB (2007). Cell surface chondroitin sulfate glycosaminoglycan in melanoma: role in the activation of pro-MMP-2 (progelatinase A). Biochem J.

[CR25] Issitt T, Bosseboeuf E, De Winter N, Dufton N, Gestri G, Senatore V, Chikh A, Randi AM, Raimondi C (2019). Neuropilin-1 controls endothelial homeostasis by regulating mitochondrial function and iron-dependent oxidative stress. Science.

[CR26] Jia XL, Li SY, Dang SS, Cheng YA, Zhang X, Wang WJ, Hughes CE, Caterson B (2012). Increased expression of chondroitin sulphate proteoglycans in rat hepatocellular carcinoma tissues. World J Gastroenterol.

[CR27] Jinno A, Park PW (2015). Role of glycosaminoglycans in infectious disease. Methods Mol Biol.

[CR28] Kastana P, Choleva E, Poimenidi E, Karamanos N, Sugahara K, Papadimitriou E (2019). Insight into the role of chondroitin sulfate E in angiogenesis. FEBS J.

[CR29] Kim JS, Werth VP (2011). Identification of specific chondroitin sulfate species in cutaneous autoimmune disease. J Histochem Cytochem.

[CR30] Klein JA, Meng L, Zaia J (2018). Deep sequencing of complex proteoglycans: a novel strategy for high coverage and site-specific identification of glycosaminoglycan-linked peptides. Mol Cell Proteomics.

[CR31] Klein J, Carvalho L, Zaia J (2018). Application of network smoothing to glycan LC-MS profiling. Bioinformatics.

[CR32] Klüppel M, Wight TN, Chan C, Hinek A, Wrana JL (2005). Maintenance of chondroitin sulfation balance by chondroitin-4-sulfotransferase 1 is required for chondrocyte development and growth factor signaling during cartilage morphogenesis. Development.

[CR33] Koike T, Izumikawa T, Tamura J, Kitagawa H (2009). FAM20B is a kinase that phosphorylates xylose in the glycosaminoglycan-protein linkage region. Biochem J.

[CR34] Koike T, Mikami T, Shida M, Habuchi O, Kitagawa H (2015). Chondroitin sulfate-E mediates estrogen-induced osteoanabolism. Sci Rep.

[CR35] Kwok JC, Warren P, Fawcett JW (2012). Chondroitin sulfate: a key molecule in the brain matrix. Int J Biochem Cell Biol.

[CR36] Lebrilla CB, Liu J, Widmalm G, Prestegard JH (2022) Oligosaccharides and Polysaccharides. In: Varki A, Cummings RD, Esko JD, et al (ed). Essentials of Glycobiology [Internet]. 4th edition. Cold Spring Harbor (NY): Cold Spring Harbor Laboratory Press; 2022. Chapter 3. Available via: https://www.ncbi.nlm.nih.gov/books/NBK579972/ 10.1101/glycobiology.4e.3

[CR37] Lindahl U, Couchman J, Kimata K, Esko JD (2017) Proteoglycans and Sulfated Glycosaminoglycans. In: Varki A, Cummings RD, Esko JD, et al (ed) Essentials of Glycobiology [Internet]. 3rd edition. Cold Spring Harbor (NY): Cold Spring Harbor Laboratory Press; 2015–2017. Available via https://www.ncbi.nlm.nih.gov/books/NBK453033/ https//doi.org/ 10.1101/glycobiology.3e.017

[CR38] Ly M, Laremore TN, Linhardt RJ (2010). Proteoglycomics: recent progress and future challenges. OMICS.

[CR39] Maeda N, Fukazawa N, Ishii M (2010). Chondroitin sulfate proteoglycans in neural development and plasticity. Front Biosci (landmark Ed).

[CR40] Mao Y, Wang S, Zhao Y, Konstantinidi A, Sun L, Ye Z, Vakhrushev SY (2021). Systematic evaluation of fragmentation methods for unlabeled and isobaric mass tag-labeled O-glycopeptides. Anal Chem.

[CR41] Meester JAN, Verstraeten A, Schepers D, Alaerts M, Van Laer L, Loeys BL (2017) Differences in manifestations of Marfan syndrome, Ehlers-Danlos syndrome, and Loeys-Dietz syndrome. Ann Cardiothorac Surg 6(6):582–594. 10.21037/acs.2017.11.03 10.21037/acs.2017.11.03 PMC5721110 29270370

[CR42] Merry CLR, Lindahl U, Couchman J, Esko JD (2022) Proteoglycans and Sulfated Glycosaminoglycans. In: Varki A, Cummings RD, Esko JD, et al (ed). Essentials of Glycobiology [Internet]. 4th edition. Cold Spring Harbor (NY): Cold Spring Harbor Laboratory Press; 2022. Chapter 17. Available via: https://www.ncbi.nlm.nih.gov/books/NBK579925/10.1101/glycobiology.4e.17

[CR43] Mikami T, Kitagawa H (2013). Biosynthesis and function of chondroitin sulfate. Biochim Biophys Acta.

[CR44] Mizumoto S, Ikegawa S, Sugahara K (2013). Human genetic disorders caused by mutations in genes encoding biosynthetic enzymes for sulfated glycosaminoglycans. J Biol Chem.

[CR45] Mizumoto S, Yamada S, Sugahara K (2015). Molecular interactions between chondroitin-dermatan sulfate and growth factors/receptors/matrix proteins. Curr Opin Struct Biol.

[CR46] Moses J, Oldberg A, Fransson LA (1999). Initiation of galactosaminoglycan biosynthesis. Separate galactosylation and dephosphorylation pathways for phosphoxylosylated decorin protein and exogenous xyloside. Eur J Biochem.

[CR47] Muenzer J (2011). Overview of the mucopolysaccharidoses. Rheumatology (oxford).

[CR48] Mun DG, Renuse S, Saraswat M, Madugundu A, Udainiya S, Kim H, Park SR, Zhao H, Nirujogi RS, Na CH, Kannan N, Yates JR, Lee SW, Pandey A (2020). PASS-DIA: A Data-Independent Acquisition Approach for Discovery Studies. Anal Chem.

[CR49] Na CH, Sharma N, Madugundu AK, Chen R, Aksit MA, Rosson GD, Cutting GR, Pandey A (2019). Integrated transcriptomic and proteomic analysis of human eccrine sweat glands identifies missing and novel proteins. Mol Cell Proteomics.

[CR50] Nandini CD, Sugahara K (2006). Role of the sulfation pattern of chondroitin sulfate in its biological activities and in the binding of growth factors. Adv Pharmacol.

[CR51] Nikitovic D, Assouti M, Sifaki M, Katonis P, Krasagakis K, Karamanos NK, Tzanakakis GN (2008). Chondroitin sulfate and heparan sulfate-containing proteoglycans are both partners and targets of basic fibroblast growth factor-mediated proliferation in human metastatic melanoma cell lines. Int J Biochem Cell Biol.

[CR52] Nikpour M, Nilsson J, Persson A, Noborn F, Vorontsov E, Larson G (2021). Proteoglycan profiling of human, rat and mouse insulin-secreting cells. Glycobiology.

[CR53] Nilsson J, Rüetschi U, Halim A, Hesse C, Carlsohn E, Brinkmalm G, Larson G (2009). Enrichment of glycopeptides for glycan structure and attachment site identification. Nat Methods.

[CR54] Noborn F, Gomez Toledo A, Sihlbom C, Lengqvist J, Fries E, Kjellén L, Nilsson J, Larson G (2015). Identification of chondroitin sulfate linkage region glycopeptides reveals prohormones as a novel class of proteoglycans. Mol Cell Proteomics.

[CR56] Oegema TR, Kraft EL, Jourdian GW, Van Valen TR (1984). Phosphorylation of chondroitin sulfate in proteoglycans from the swarm rat chondrosarcoma. J Biol Chem.

[CR57] Orend G, Chiquet-Ehrismann R (2000). Adhesion modulation by antiadhesive molecules of the extracellular matrix. Exp Cell Res.

[CR58] Oxvig C, Haaning J, Højrup P, Sottrup-Jensen L (1994). Location and nature of carbohydrate groups in proform of human major basic protein isolated from pregnancy serum. Biochem Mol Biol Int.

[CR59] Paganini C, Costantini R, Superti-Furga A, Rossi A (2019). Bone and connective tissue disorders caused by defects in glycosaminoglycan biosynthesis: a panoramic view. FEBS J.

[CR60] Perrimon N, Bernfield M (2001). Cellular functions of proteoglycans–an overview. Semin Cell Dev Biol.

[CR61] Peterson SL, Husnain M, Pollack T, Pimentel A, Loaiza-Bonilla A, Westendorf-Overley C, Ratermann K, Anthony L, Desimone P, Goel G, Kudrimoti M, Dineen S, Tzeng CD, Hosein PJ (2018) Neoadjuvant Nab-paclitaxel and Gemcitabine in Borderline Resectable or Locally Advanced Unresectable Pancreatic Adenocarcinoma in Patients Who Are Ineligible for FOLFIRINOX. Anticancer Res. 38(7):4035–4039. 10.21873/anticanres.12692 10.21873/anticanres.12692 PMC9375526 29970528

[CR62] Prydz K, Dalen KT (2000). Synthesis and sorting of proteoglycans. J Cell Sci.

[CR63] Qiao D, Meyer K, Friedl A (2016). Glypican-1 stimulates Skp2 autoinduction loop and G1/S transition in endothelial cells. J Biol Chem.

[CR64] Riley NM, Malaker SA, Driessen MD, Bertozzi CR (2020). Optimal Dissociation Methods Differ for N- and O-Glycopeptides. J Proteome Res.

[CR65] Ryu CS, Klein K, Zanger UM (2017). Membrane Associated Progesterone Receptors: Promiscuous Proteins with Pleiotropic Functions - Focus on Interactions with Cytochromes P450. Front Pharmacol.

[CR66] Saraswat M, Garapati K, Mun DG, Pandey A (2021). Extensive heterogeneity of glycopeptides in plasma revealed by deep glycoproteomic analysis using size-exclusion chromatography. Mol Omics.

[CR91] Schittek B (2012) The multiple facets of dermcidin in cell survival and host defense. J Innate Immun 4(4):349-360. 10.1159/000336844 10.1159/000336844 PMC6741627 22455996

[CR67] Scuruchi M, Potì F, Rodríguez-Carrio J, Campo GM, Mandraffino G (2020). Biglycan and atherosclerosis: Lessons from high cardiovascular risk conditions. Biochim Biophys Acta Mol Cell Biol Lipids.

[CR68] Seno N, Sekizuka E (1978). Structure of linkage region between chondroitin polysulfates and peptides. J Biochem.

[CR69] Shikata Y, Hayashi Y, Yoshimatsu K, Ohya Y, Seto T, Fukushima K, Yoshida Y (1993). Pro-major basic protein has three types of sugar chains at the pro-portion. Biochim Biophys Acta.

[CR70] Shintani Y, Takashima S, Asano Y, Kato H, Liao Y, Yamazaki S, Tsukamoto O, Seguchi O, Yamamoto H, Fukushima T, Sugahara K, Kitakaze M, Hori M (2006). Glycosaminoglycan modification of neuropilin-1 modulates VEGFR2 signaling. EMBO J.

[CR71] Siebert JR, Conta Steencken A, Osterhout DJ (2014). Chondroitin sulfate proteoglycans in the nervous system: inhibitors to repair. Biomed Res Int.

[CR72] Sisu E, Flangea C, Serb A, Zamfir AD (2011). Modern developments in mass spectrometry of chondroitin and dermatan sulfate glycosaminoglycans. Amino Acids.

[CR74] Stephenson EL, Mishra MK, Moussienko D, Laflamme N, Rivest S, Ling CC, Yong VW (2018) Chondroitin sulfate proteoglycans as novel drivers of leucocyte infiltration in multiple sclerosis. Brain. 141(4):1094–1110. https:doi.org/10.1093/brain/awy033 10.1093/brain/awy033 PMC5888970 29506186

[CR75] Stern EL, Lindahl B, Rodén L (1971) The linkage of dermatan sulfate to protein. II. Monosaccharide sequence of the linkage region. J Biol Chem. 246(18):5707–5715 5096091

[CR76] Stringer SE (2006). The role of heparan sulphate proteoglycans in angiogenesis. Biochem Soc Trans.

[CR77] Teesalu T, Sugahara KN, Kotamraju VR, Ruoslahti E (2009). C-end rule peptides mediate neuropilin-1-dependent cell, vascular, and tissue penetration. Proc Natl Acad Sci U S A.

[CR78] Ten Dam GB, van de Westerlo EM, Purushothaman A, Stan RV, Bulten J, Sweep FC, Massuger LF, Sugahara K, van Kuppevelt TH (2007). Antibody GD3G7 selected against embryonic glycosaminoglycans defines chondroitin sulfate-E domains highly up-regulated in ovarian cancer and involved in vascular endothelial growth factor binding. Am J Pathol.

[CR79] Theocharis AD, Tsolakis I, Tzanakakis GN, Karamanos NK (2006). Chondroitin sulfate as a key molecule in the development of atherosclerosis and cancer progression. Adv Pharmacol.

[CR80] Toledo AG, Pihl J, Spliid CB, Persson A, Nilsson J, Pereira MA, Gustavsson T, Choudhary S, Oo HZ, Black PC, Daugaard M, Esko JD, Larson G, Salanti A, Clausen TM (2020). An affinity chromatography and glycoproteomics workflow to profile the chondroitin sulfate proteoglycans that interact with malarial VAR2CSA in the placenta and in cancer. Glycobiology.

[CR81] Tone Y, Pedersen LC, Yamamoto T, Izumikawa T, Kitagawa H, Nishihara J, Tamura J, Negishi M, Sugahara K (2008). 2-o-phosphorylation of xylose and 6-o-sulfation of galactose in the protein linkage region of glycosaminoglycans influence the glucuronyltransferase-I activity involved in the linkage region synthesis. J Biol Chem.

[CR82] Tran AP, Sundar S, Yu M, Lang BT, Silver J (2018). Modulation of receptor protein tyrosine phosphatase sigma increases chondroitin sulfate proteoglycan degradation through cathepsin b secretion to enhance axon outgrowth. J Neurosci.

[CR83] Vainauskas S, Duke RM, McFarland J, McClung C, Ruse C, Taron CH (2016). Profiling of core fucosylated N-glycans using a novel bacterial lectin that specifically recognizes α1,6 fucosylated chitobiose. Sci Rep.

[CR84] Wang W, Shi L, Yong Q, Li F (2020). Research and application of chondroitin sulfate/dermatan sulfate-degrading enzymes. Front Cell Dev Biol.

[CR90] Wei Poh Zhong, Heng Gan Chin, Lee Eric J, Guo Suxian, Yip George W, Lam Yulin (2015). Divergent synthesis of chondroitin sulfate disaccharides and identification of sulfate motifs that inhibit triple negative breast cancer. Sci Rep.

[CR85] Wei J, Hu M, Huang K, Lin S, Du H (2020). Roles of proteoglycans and glycosaminoglycans in cancer development and progression. Int J Mol Sci.

[CR86] Wells L, Vosseller K, Cole RN, Cronshaw JM, Matunis MJ, Hart GW (2002). Mapping sites of O-GlcNAc modification using affinity tags for serine and threonine post-translational modifications. Mol Cell Proteomics.

[CR87] Wen J, Xiao J, Rahdar M, Choudhury BP, Cui J, Taylor GS, Esko JD, Dixon JE (2014). Xylose phosphorylation functions as a molecular switch to regulate proteoglycan biosynthesis. Proc Natl Acad Sci U S A.

[CR88] Zhou ZH, Karnaukhova E, Rajabi M, Reeder K, Chen T, Dhawan S, Kozlowski S (2014). Oversulfated chondroitin sulfate binds to chemokines and inhibits stromal cell-derived factor-1 mediated signaling in activated T cells. PLoS ONE.

[CR89] Zhuo L, Salustri A, Kimata K (2002). A physiological function of serum proteoglycan bikunin: the chondroitin sulfate moiety plays a central role. Glycoconj J.

