# Supplementary material for: Mass spectrometric analysis of chondroitin sulfate-linked peptides
Source: J Proteins Proteom. 2022 Oct 2;13(4):187–203. doi: 10.1007/s42485-022-00092-3 (PMC9526814; doi:10.1007/s42485-022-00092-3)
Supplement: Supplementary file 1 — Supplementary file1 (PDF 144 KB) [file 42485_2022_92_MOESM1_ESM.pdf]

Supplementary Information

Mass spectrometric analysis of chondroitin sulfate-linked peptides

Madan Gopal Ramarajan[1,2,3,4#], Mayank Saraswat[1,2,3#], Rohit Budhraja[1], Kishore Garapati[1,2,3,4], Kimiyo Raymond[5], Akhilesh Pandey[1,6*]

[1]Department of Laboratory Medicine and Pathology, Mayo Clinic, Rochester, MN 55905, USA
[2]Institute of Bioinformatics, International Technology Park, Bangalore, 560066 India
[3]Manipal Academy of Higher Education (MAHE), Manipal, 576104, Karnataka, India
[4]Center for Molecular Medicine, National Institute of Mental Health and Neurosciences (NIMHANS), Hosur Road, Bangalore 560 029, India
[5]Biochemical Genetics Laboratory, Department of Laboratory Medicine and Pathology, Mayo Clinic, Rochester, MN 55905, USA
[6]Center for Individualized Medicine, Mayo Clinic, Rochester, MN 55905, USA

[#]Co-first author. These authors contributed equally to this work

[*]Correspondence
Akhilesh Pandey, M.D., Ph.D.
Professor
Department of Laboratory Medicine and Pathology, Mayo Clinic
200 First ST SW, Rochester, MN 55905
Phone: 507-293-9564
E-mail: pandey.akhilesh@mayo.edu
ORCID ID: 0000-0001-9943-6127

Supplementary table S1: List of glycan linker compositions identified on chondroitin sulfate linked glycopeptides

Code	Glycan composition	Glycan sequence
CS01	Xyl:1; a,en-Hex:1; Hex:2; a-Hex:1; HexNAc:1	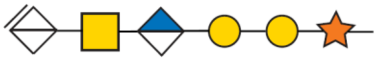
CS02	Xyl:1; a,en-Hex:1; Hex:2; a-Hex:1; HexNAc(S):1	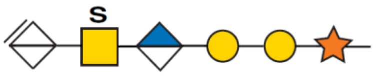
CS03	a,en-Hex:1; Hex:2; a-Hex:1; HexNAc:1; XylP:1	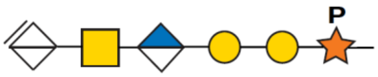
CS04	a,en-Hex:1; Hex:2; a-Hex:1; XylP:1; HexNAc(S):1	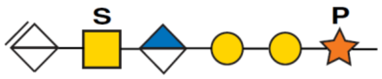
CS05	Xyl:1; a,en-Hex:1; Hex:1; a-Hex:1; HexS:1; HexNAc(S):1	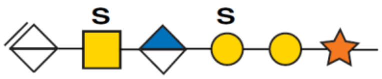
CS06	Xyl:1; a,en-Hex:1; Hex:2; a-Hex:1; HexNAc(S):1; Neu5Ac:1	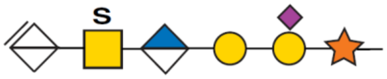
CS07	Xyl:1; Fuc:1; a,en-Hex:1; Hex:2; a-Hex:1; HexNAc(S):1	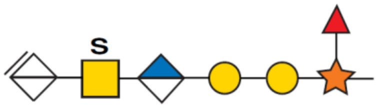
CS08	a,en-Hex:1; Hex:1; a-Hex:1; XylP:1; HexS:1; HexNAc(S):1	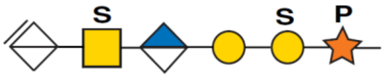
CS09	Xyl:1; a,en-Hex:1; a-Hex:1; HexS:2; HexNAc(S):1	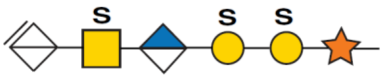
CS10	a,en-Hex:1; Hex:2; a-Hex:1; XylP:1; HexNAc(S):1; Neu5Ac:1	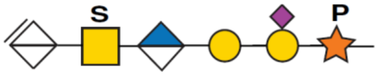
CS11	Xyl:1; a,en-Hex:1; Hex:1; a-Hex:1; HexS:1; HexNAc(S):1; Neu5Ac:1	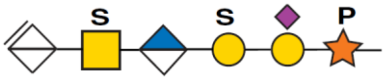
CS12	Xyl:1; Fuc:1; a,en-Hex:1; Hex:1; a-Hex:1; HexS:1; HexNAc(S):1	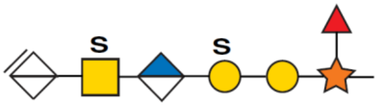
CS17	Fuc:1; a,en-Hex:1; Hex:2; a-Hex:1; HexNAc:1; XylP:1	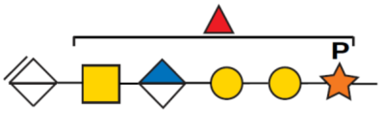
CS30	Xyl:1; a,en-Hex:1; Hex:2; a-Hex:1; HexNAc:1; Neu5Ac:1	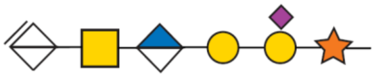
